# Emotion recognition and self-versus-other referential learning in mood disorders and schizophrenia

**DOI:** 10.1192/j.eurpsy.2025.1681

**Published:** 2025-08-26

**Authors:** C.-D. Chiu

**Affiliations:** Department of Psychology, Chinese University of Hong Kong, North Territory, Hong Kong

## Abstract

**Introduction:**

Patients of depression and psychotic disorders are often troubled by unsatisfactory interpersonal relationships. While an inability to maintain a stable sense of self restricts one’s understanding another’s emotional state, whether disrupted self-versus-other referential processing is a transdiagnostic predictor of increased emotion misreading across diagnostic groups has not been explicated.

**Objectives:**

We tested whether weakened differential learning between self and other may account for impoversihed emotion recognition accross mood and psychotic disorders.

**Methods:**

Inpatients admitted for major depressive disorder (MDD), bipolar disorder (BD), and schizophrenia (SCZ; ns = 59, 32, and 43) and 40 healthy controls were recruited. Aside from ratings of depressive and schizophrenic symptoms by psychiatrists, participants were assessed on self- versus other- referential learning, emotion recognition, emotion sharing.

**Results:**

Regression analysis indicates lower effectiveness of self-other tagging to be a predictor independent from symptom severity for increased emotion misrecognition across MDD, BD and SCZ (*F*(8, 160) = 8.52, *p* < 0.001). Clinical groups showed lower accuracy for other-referential recall and emotion recognition, but comparable emotion sharing and self-prioritization to healthy controls.

**Image:**

**Figure:**
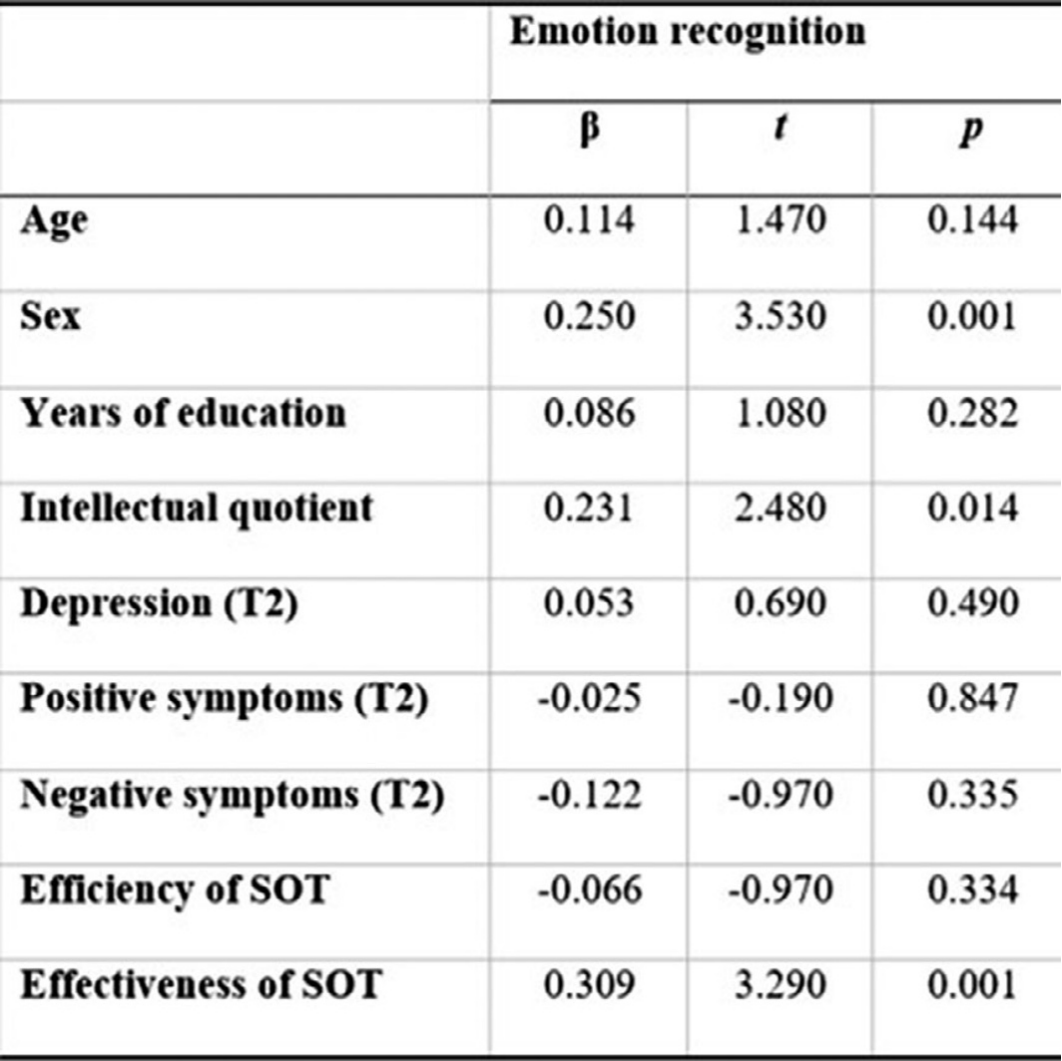

**Conclusions:**

Heightened emotion misrecognition in MDD, CD, and SCZ patients can be traced back to the weakened ability in coordinating self- and other-representations according to task-demands. Future examinations on whether interventions on brain regions pertaining to self-versus-other learning might enhance emotion recognition in different patient groups would be clinically relevant.

**Disclosure of Interest:**

None Declared

